# A Genome-Wide Screen for Genetic Variants That Modify the Recruitment of REST to Its Target Genes

**DOI:** 10.1371/journal.pgen.1002624

**Published:** 2012-04-05

**Authors:** Rory Johnson, Nadine Richter, Gireesh K. Bogu, Akshay Bhinge, Siaw Wei Teng, Siew Hua Choo, Lise O. Andrieux, Cinzia de Benedictis, Ralf Jauch, Lawrence W. Stanton

**Affiliations:** 1 Stem Cell and Developmental Biology Group, Genome Institute of Singapore, Singapore, Singapore; 2 Laboratory of Structural Biochemistry, Genome Institute of Singapore, Singapore, Singapore; 3 Bioinformatics and Genomics Group, Centre for Genomic Regulation (CRG) and UPF, Barcelona, Spain; 4 Department of Biological Sciences, National University of Singapore, Singapore, Singapore; Friedrich Miescher Institute for Biomedical Research, Switzerland

## Abstract

Increasing numbers of human diseases are being linked to genetic variants, but our understanding of the mechanistic links leading from DNA sequence to disease phenotype is limited. The majority of disease-causing nucleotide variants fall within the non-protein-coding portion of the genome, making it likely that they act by altering gene regulatory sequences. We hypothesised that SNPs within the binding sites of the transcriptional repressor REST alter the degree of repression of target genes. Given that changes in the effective concentration of REST contribute to several pathologies—various cancers, Huntington's disease, cardiac hypertrophy, vascular smooth muscle proliferation—these SNPs should alter disease-susceptibility in carriers. We devised a strategy to identify SNPs that affect the recruitment of REST to target genes through the alteration of its DNA recognition element, the RE1. A multi-step screen combining genetic, genomic, and experimental filters yielded 56 polymorphic RE1 sequences with robust and statistically significant differences of affinity between alleles. These SNPs have a considerable effect on the the functional recruitment of REST to DNA in a range of in vitro, reporter gene, and in vivo analyses. Furthermore, we observe allele-specific biases in deeply sequenced chromatin immunoprecipitation data, consistent with predicted differenes in RE1 affinity. Amongst the targets of polymorphic RE1 elements are important disease genes including *NPPA*, *PTPRT*, and *CDH4*. Thus, considerable genetic variation exists in the DNA motifs that connect gene regulatory networks. Recently available ChIP–seq data allow the annotation of human genetic polymorphisms with regulatory information to generate prior hypotheses about their disease-causing mechanism.

## Author Summary

Common human diseases such as cancer, heart disease, or epilepsy have a genetic component that predisposes particular individuals to suffer from them. Huge sums have been invested to map the regions of the human genome where small DNA variations, or SNPs (“single-nucleotide polymorphisms”), determine the probability of developing these diseases. A major problem with this approach, however, is that, once the culprit SNPs are discovered, we know very little about *how* they cause disease—which is critical if we are to use this information to develop drugs and therapies. In this study, we demonstrate a new approach, employing functional maps of the human genome that have recently been published. We begin with regions of the genome recognised by a gene repressor protein—REST—that is involved in a number of important human diseases. Using information on where REST binds in the human genome, we predict and validate common DNA variations that increase or decrease this binding. By affecting how much REST is recruited to important genes, these variations may predispose or protect individuals from a number of diseases. Studies like this show how we can use genomic information to gain a deeper understanding of the genetics behind human disease.

## Introduction

Genetic factors underlie the distinct phenotypic traits and disease susceptibilities that are observed between human individuals and populations [1]. Huge resources have been allocated to mapping genetic variants - particularly the smallest, single nucleotide variants (SNPs) - that correlate with numerous human traits, including obesity, blood pressure, and schizophrenia [2]. While these projects have uncovered several thousand disease risk variants, such genome-wide association studies suffer from a major drawback: they provide little prior information or hypothesis on the mechanism by which an associated SNP causes the observed phenotype. Such mechanistic insight will be crucial if genetic information is to lead to therapeutic strategies to treat genetic diseases. Given that the majority of functional SNPs lie outside protein-coding regions and are likely to act by altering gene regulatory sequences, the genome-wide annotation of regulatory SNPs represents a promising avenue for the comprehensive prediction of SNP mechanisms-of-action [3].

It is likely that many non-coding SNPs lie within regulatory DNA motifs, altering their affinity for transcription factors and thus also altering the expression levels of genes *cis*-targeted by those motifs. In agreement with this, variation in gene transcript levels has been observed between individuals, and these differences correlated with non-coding SNP alleles [4]. However, it has until recently been difficult to establish mechanistic consequences of SNPs at a genome-wide level because (a) most transcriptional regulatory motifs can not be confidently identified based on DNA sequence alone, and (b) genome-wide experimental maps of transcription factor binding have not been available. Nevertheless, a number of validated cases do exist where a SNP has been shown to alter the regulation of a nearby gene: for example, a SNP in the *VEGF* promoter converts non-functional DNA into a p53 response element in 6% of genotyped individuals [5]. Indeed, it has been shown in a small number of cases that regulatory SNPs that create or disrupt transcriptional regulatory motifs can result in genetic disease [6], [7]. Clearly, the ultimate aim must be to comprehensively annotate regulatory SNPs in human populations. In recent years, a number of studies have employed human genome sequence to systematically hunt for regulatory SNPs lying in the predicted binding sites of particular transcription factors, notably NRF2 [8] and USF1 [9]. Unfortunately, relying on genome sequence analysis alone to discover transcription factor binding sites is plagued by high false positive rates, especially for the majority of factors that recognise short or degenerate DNA motifs. Recently, the development of high-throughput sequencing has offered a solution to this problem, by enabling the genome-wide experimental mapping of transcription factor binding sites using the ChIPseq method [10]. Thus it is possible, for the first time, to make highly accurate genomic annotations of regulatory SNPs affecting transcriptional regulation.

For practical reasons, we propose that a genome-wide search for regulatory SNPs should select a system with the following properties: (a) the transcriptional regulatory sequence should be clearly recognisable by motif analysis; (b) there must be an experimentally-derived, genome-wide map for the transcription factor in question; and (c) that transcription factor should have known roles in human disease. In this study, we describe such a study on the transcriptional repressor, REST (RE1-Silencing Transcription Factor). This essential zinc finger protein represses numerous genes in various developmental stages [11] and adult tissues [12]. Importantly, changes in the effective nuclear levels of REST are pathological in cancer [13], [14], as well as cardiac hypertrophy [15], vascular smooth muscle proliferation [12], ischaemia [16] and Huntington's disease [17]. Consequently, this system is a promising drug target [18]. REST is recruited to target genes by a long, characteristic motif - the RE1 (Repressor Element 1) - which can be identified with high confidence using motif-finding methods [19]. Finally, REST was the first factor whose binding sites were mapped by ChIPseq (chromatin immunoprecipitation followed by high throughput sequencing) in the human genome [10], and since then a number of additional datasets in various cell types have become available. In this paper, we hypothesise that SNP variants in RE1 motifs can affect the in vivo recruitment of REST to target genes, resulting in differential gene repression, and altering the severity or frequency with which individuals are affected by the abovementioned diseases.

Overall, RE1 motifs are depleted for SNPs, indicating that these motifs are under negative evolutionary selection and that in general, polymorphisms that affect the affinity of RE1 motifs have a deleterious effect on their carrier [20]. Nevertheless, 8% of RE1s contain at least one annotated SNP. In order to identify phenotypically important SNPs that act through the modulation of mRNA levels of REST target genes, we designed a multi-step screen to discover polymorphic RE1 sites (henceforth referred to as “pRE1s”) and measured allelic affinity differences in vitro. We present analysis of 56 SNPs that are predicted to strongly affect the binding affinity of REST to its target genes in vivo, making them candidate modulators of disease susceptibility.

## Results

**Figure 1:**
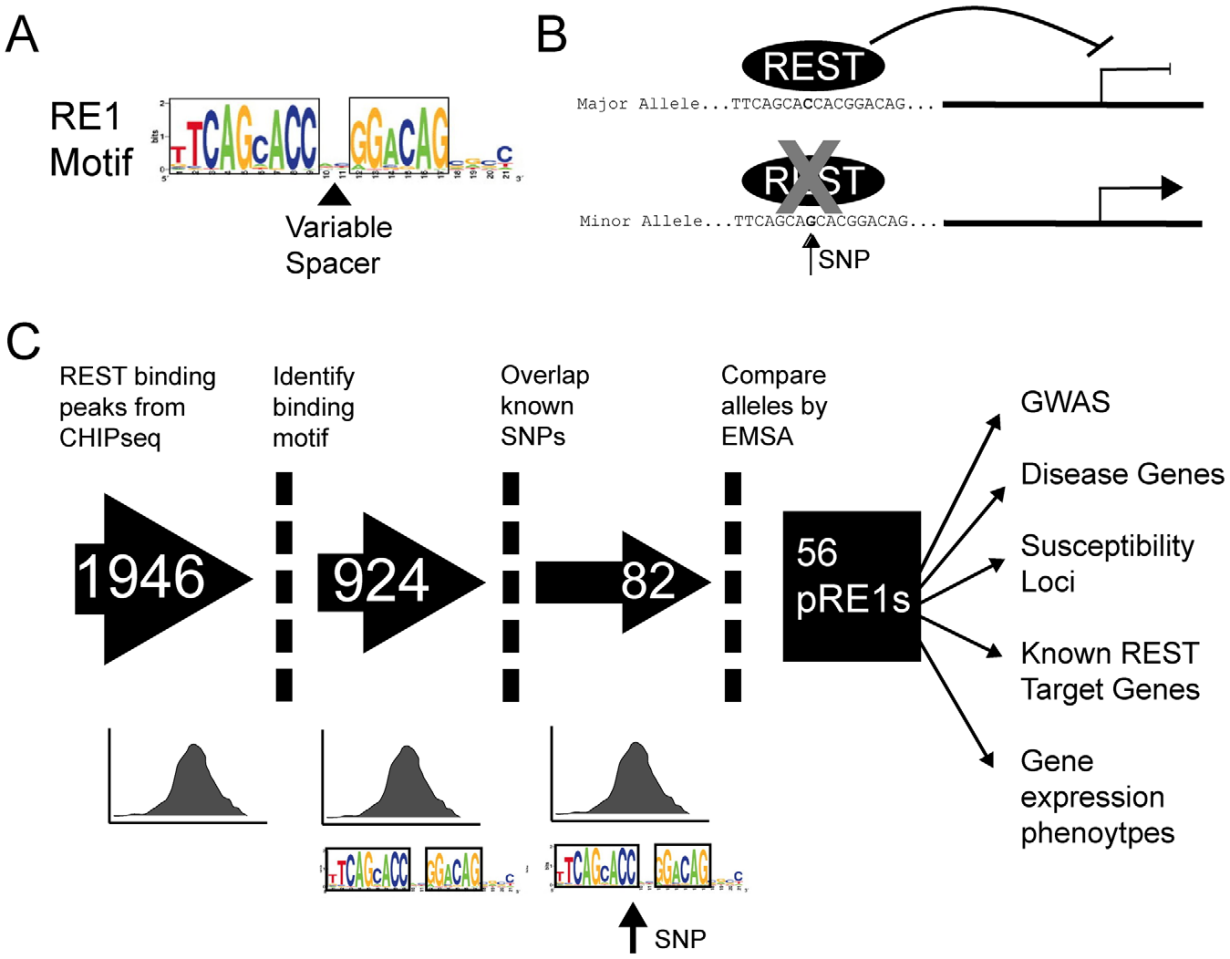
Experimental pipeline to discover SNPs that affect gene repression by REST. (A) The structure of the RE1 motif, illustrating its two strongly constrained half sites and weakly constrained spacer and 3′ regions. The spacer region may have “canonical” size of two nucleotides, or other “non-canonical” sizes. (B) Cartoon illustrating the hypothetical effect of a SNP in an RE1 element. In the upper panel, the Major (ie more frequent) allele contains a high-affinity RE1 sequence that strongly recruits REST, resulting in target gene repression. The presence of the SNP reduces REST binding affinity, and results in an increase in target gene transcription. (C) The flowchart illustrates the pipeline employed in this study to discover pRE1 SNPs.

Our aim was to curate the set of genetic polymorphisms that affect the function of REST by altering the affinity of its cognate genomic DNA recognition elements, RE1s (Figure 1B). The RE1 consists of left and right half sites of 9 and 6 basepairs, respectively, separated by a spacer of two nucleotides (“canonical motif”) (Figure 1A). It was recently shown that an additional population of binding sites contain “noncanonical” RE1 motifs having more or less spacer nucleotides [10]. Genome-wide studies have also identified REST binding regions containing one or other RE1 half site, or indeed no identifiable RE1 motif at all, although it remains unclear to what extent REST recruitment to these regions depends on specific DNA recognition or whether other factors are involved such as recruitment by another protein complex [10], [21]. We obtained an experimentally determined set of REST binding regions in a human cell line, determined by the high-throughput sequencing-based ChIPseq method [10] (Figure 1C). This dataset, containing 1946 binding sites, is purely experimental and does not explicitly contain information about the DNA motif responsible for REST binding at each location. Thus, we passed all ChIPseq sites through a bioinformatic motif finding filter, retaining only those 924 instances containing an identifiable RE1 element (Figure 1C). Importantly, we included RE1 motifs having both canonical and non-canonical spacer configurations, but excluded all other sites lacking both RE1 half sites within 12 nt of each other. This validated RE1 motif set was next cross referenced to the set of all known single nucleotide variants from dbSNP129, yielding a set of 86 SNPs lying within 82 RE1 motifs (3 RE1s each had 2 SNPs at distinct locations), of which 52/30 were canonical/noncanonical, respectively. Henceforth we refer to these RE1 motifs as “Polymorphic RE1s” or pRE1s, and for convenience we number them 1–82. The complete set of pRE1s can be found in File S1. We hypothesised that these SNPs should affect the regulation of these genes by REST in vivo, by altering their binding affinity (Figure 1B).

**Figure 2:**
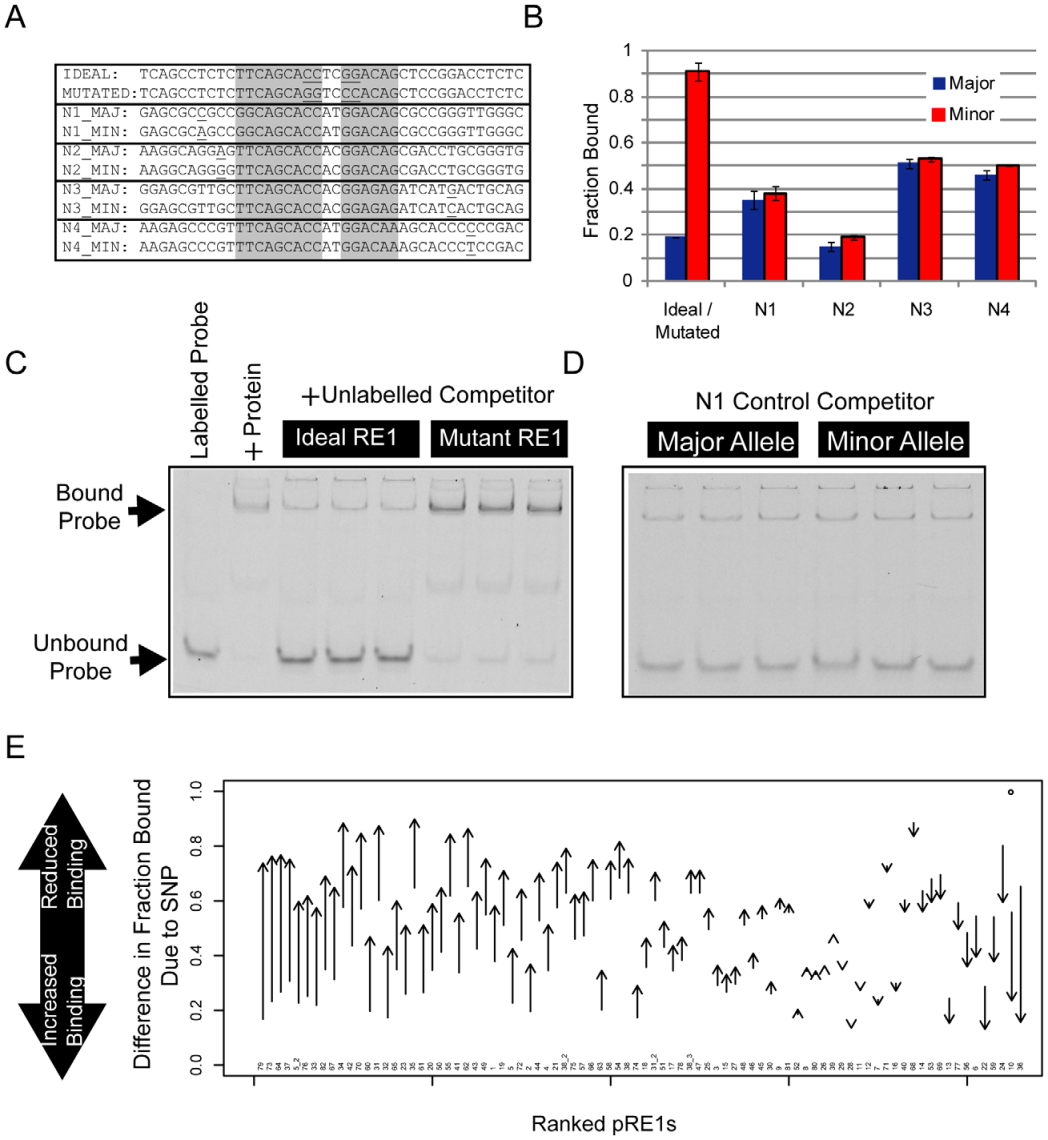
Electrophoretic mobility shift assay to measure affinity differences between RE1 alleles. To measure RE1 affinity in vitro, we employed a competition EMSA method. We tested the ability of unlabelled competitor sequences to compete for REST binding with a fluorescently labelled DNA probe. (A) Various control oligonucleotides were used to validate the sensitivity and selectivity of the comparative EMSA assay. The Ideal RE1 motif is a high affinity synthetic sequence we used previously [20]. By swapping two highly conserved dinucleotides in the sequence, the affinity of the Ideal RE1 can be completely abolished (Mutated RE1). We also designed four pairs of RE1 alleles (N1-4), where SNPs lie outside the RE1 half sites, and thus would not be expected to alter binding affinity. (B) The results of control EMSAs are shown, where replicate EMSA gels have been quantitated and plotted. The data are displayed in units of Fraction Bound (see Materials and Methods), where a low Fraction Bound value indicates high binding affinity, and vice versa. Example raw data are shown in panels: (C) Ideal/Mutated RE1s and (D) N1 RE1s. (E) Summary results of EMSA for all pRE1s in this study. The y-axis plots the difference in Fraction Bound between Major and Minor alleles, where the arrow begins at the value for Major, and ends at Minor. All RE1s are ranked by their change in Fraction Bound.

The final component of the screen was an in vitro binding assay to define the biochemical effect of SNP polymorphism on RE1 affinity. We used the competition electrophoretic mobility shift assay that we successfully employed previously [20] to measure REST binding to pRE1 variants. In this assay, purified recombinant REST protein is mixed with a high affinity, fluorescently-labelled RE1 oligonucleotide probe. The affinity of various putative RE1 sequences can then be measured by their ability to compete the protein∶probe interaction. A comparison of the bound and unbound probe concentrations yields a measure – “Fraction Bound” - that approaches 1 for a competitor sequence that cannot bind REST, and approaches 0 for a sequence that strongly binds REST. We refer to the more/less frequent SNP allele as the “Major”/“Minor” variants, respectively. Experiments were carried out for multiple replicates, allowing us to discern differential affinity with statistical significance. We optimized the EMSA conditions by maximising the Fraction Bound difference between idealised (high affinity) and mutated (low affinity) synthetic RE1 competitor sequences. In this way, we were able to capture allelic affinity differences between high-quality RE1 sequences and their mutants (Figure 2). First, we validated the screening approach using a series of control RE1 sequences (designated N1-N4), containing SNPs outside the core REST recognition motif. None of these peripheral SNPs had a detectable effect on REST affinity (Figure 2A, 2B, 2D).

**Figure 3:**
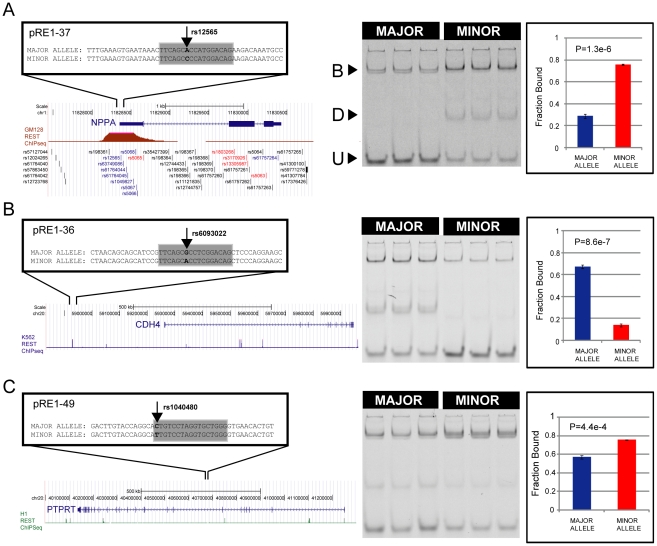
SNPs may increase or decrease affinity of RE1s. Examples of SNPs that decrease (A) and increase (B) the affinity of an RE1 sequence. On the left are diagrams of the genomic location of polymorphic RE1s, their target genes and the REST ChIPseq read density taken from ENCODE data. On the right is corresponding quantitative EMSA data. In (A), the well-studied RE1 that lies within the 3′ UTR of the *NPPA* gene contains the SNP rs12565 that strongly decreases its affinity for REST. In (B), the RE1 lying distally upstream of the *CDH4* gene contains the SNP rs6093022 that strongly increases its affinity. (C) The SNP rs1040480 within an intron of *PTPRT* reduces the affinity of REST. B = Bound complex of REST with probe; U = Unbound probe; D = Degradation product. The latter band represents a fraction of purified REST protein that is partially degraded, as was observed previously [20].

Next, we measured REST binding to the major and minor alleles of the 82 pRE1s discovered in the bioinformatic screen (Figure 2E). Fifty six of these exhibited significant allelic affinity differences for REST. In most cases the minor variant was found to bind less strongly to REST (46/56), while for the remaining eight cases the minor variant bound more strongly (the likely reason for this discrepancy is covered in the Discussion). This number was found by imposing a minimum difference in Fold Change between alleles of 0.1. If we simply use a statistical cutoff (P
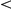
0.05, uncorrected for multiple testing) then we find 70 pRE1s with significantly different affinity between alleles. These data are summarised in Figure 2E, Tables 1 and 2. Examples of three pRE1s are shown in Figure 3. The SNP rs12565 within the pRE1 of the *NPPA* 3′ UTR has a minor allele that strongly reduces its binding affinity (Figure 3A). Conversely, the SNP rs6093022 in the pRE1 upstream of the *CDH4* gene strongly increases its affinity for REST (Figure 3B). Finally, the minor allele of the SNP rs1040480, located in pRE1-49 within an intron of the tumour-suppressor *PTPRT* [22], reduces the affinity of REST (Figure 3C).

**Table 1:** Polymorphic RE1s where the minor allele has reduced affinity.

pRE1 ID	Location	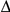 FB	SNP ID	Frequency	Gene	Dist(bp)	Disease Association
pRE1-79	chr4:26728358	0.55	rs16878854	CEU*	STIM2	256947	
pRE1-73	chr16:88422513	0.53	rs1079556	ND	SPIRE2	105	
pRE1-64	chr11:129340555	0.50	rs12802622	CEU JPT YRI	PRDM10	−17828	
pRE1-37	chr1:11828357	0.47	rs12565	ND	NPPA	2070	Familial atrial fibrillation
pRE1-67	chr16:88422513	0.37	rs2376876	ND	SPIRE2	105	
pRE1-76	chr19:45318825	0.37	rs12461190	-	ZNF780A	−30140	
pRE1-33	chr15:81129125	0.35	rs2303988	ND	CPEB1	−15342	Chronic lymphocytic leukemia
pRE1-82	chr9:130021147	0.32	rs36009729	ND	DNM1	15663	Nicotine dependence
pRE1-34	chr3:13901643	0.30	rs12629044	CEU*	WNT7A	−5024	Limb deformity
pRE1-61	chr22:32763132	0.28	rs5994827	ND	LARGE	−116722	Muscular dystrophy
pRE1-57	chr8:137199432	0.27	rs62526589	CEU*	KHDRBS3	660550	Medulloblastoma, childhood absence epilepsy
pRE1-65	chr11:11345063	0.27	rs16909532	-	GALNTL4	255062	
pRE1-70	chr10:94137238	0.27	rs11594670	-	MARCH5	96338	
pRE1-5-2	chr14:90031546	0.26	rs12893572	ND	CALM1	98420	Osteoarthritis
pRE1-20	chr19:11320989	0.26	rs12984429	ND	TMEM205	−3267	
pRE1-38-2	chr2:91125788	0.26	rs2599127	ND	RPIA	2353497	Ribose 5-phosphate isomerase deficiency
pRE1-31-1	chr5:1476347	0.26	rs423091	ND	SLC6A3	20012	Attention deficit hyperactivity disorder, major affective disorder, tobacco addiction
pRE1-23	chr17:23114277	0.25	rs28944187	ND	NOS2	37405	Hypertension, malaria infection
pRE1-32	chr1:241249144	0.25	rs61823647	ND	CEP170	236102	
pRE1-43	chr19:38576373	0.24	rs58865132	ND	CEBPG	19924	Acute myeloid leukemia
pRE1-35	chr16:47121638	0.24	rs6500392	CEU*	N4BP1	79983	
pRE1-60	chr19:4720717	0.23	rs28713481	ND	PGSF3	600	
pRE1-5-1	chr14:90031546	0.22	rs12893721	ND	CALM1	98420	Osteoarthritis
pRE1-50	chr7:64922879	0.22	rs62469938	ND	VKORC1L1	−52880	
pRE1-62	chr3:10153511	0.22	rs6796538	CEU*	VHL	−4808	Erythrocytosis, pheochromocytoma, renal cell carcinoma
pRE1-55	chr20:61747214	0.21	rs6062472	ND	STMN3	8012	
pRE1-42	chr20:43577393	0.20	rs6130854	ND	WFDC6	24155	
pRE1-72	chr15:75894622	0.19	rs55805135	ND	LINGO1	−182858	Essential tremor
pRE1-19	chr22:28204664	0.19	rs59221441	ND	NEFH	−1555	Amyotrophic lateral sclerosis
pRE1-49	chr20:40686144	0.19	rs1040480	CHB CEU JPT YRI	PTPRT	565643	Various cancers

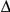
FB: Change in Fraction Bound value, FB
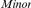
−FB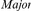; Frequency indicates Hapmap populations where the Minor SNP allele occurs at 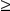
5% (ND-Not determined, -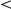
5% in all populations)(Note: genotype data come from Hapmap, except for genotyping carried out on Hapmap CEU set in this study, denoted CEU*); Dist: Distance from RE1 to gene transcriptional start site (negative indicate upstream). Known REST target genes are underlined - see File S1 for more information.

**Table 2:** Polymorphic RE1s where the minor allele has increased affinity.

pRE1 ID	Location	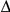 FB	SNP ID	Frequency	Gene	Dist(bp)	Disease Association
pRE1-36	chr20:58929675	−0.53	rs6093022	CEU*	CDH4	−331290	Colorectal cancer, gastric cancer
pRE1-9	chr18:39881775	−0.30	rs8092075	CEU*	SETBP1	−633086	Acute myeloid leukemia
pRE1-24	chr8:143374761	−0.25	rs28396985	CEU*	TSNARE1	107683	
pRE1-59	chr9:118157319	−0.16	rs11356865	ND	PAPPA	201427	Cardiac disease, birthweight
pRE1-6	chr10:83728154	−0.16	rs11192022	ND	NRG3	103077	Schizophrenia
pRE1-22	chr22:25675371	−0.12	rs5761858	CEU	MIAT	272933	Myocardial infarction
pRE1-69	chr22:33141085	−0.12	rs9621935	ND	LARGE	−494675	Muscular dystrophy
pRE1-56	chr4:3840072	−0.11	rs3979	CEU*	ADRA2C	101978	Congestive heart failure

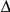
FB: Change in Fraction Bound value, FB
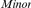
−FB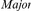; Frequency indicates Hapmap populations where the Minor SNP allele occurs at 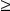
5% (ND-Not determined, -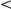
5% in all populations)(Note: genotype data come from Hapmap, except for genotyping carried out on Hapmap CEU set in this study, denoted CEU*); Dist: Distance from RE1 to gene transcriptional start site (negative indicate upstream). Known REST target genes are underlined - see File S1 for more information.

We sought to find additional evidence that the annotated target genes in this study are indeed regulated by REST. We compared the 78 predicted REST target genes from this study to recently published siREST microarray expression data [23], and found that at least 11 genes in this study respond to REST knockdown and thus may be considered to be bona fide targets (Table 1, Table 2, File S1). These include *NPPA* and *CDH4* genes mentioned above [23], [12].

**Figure 4:**
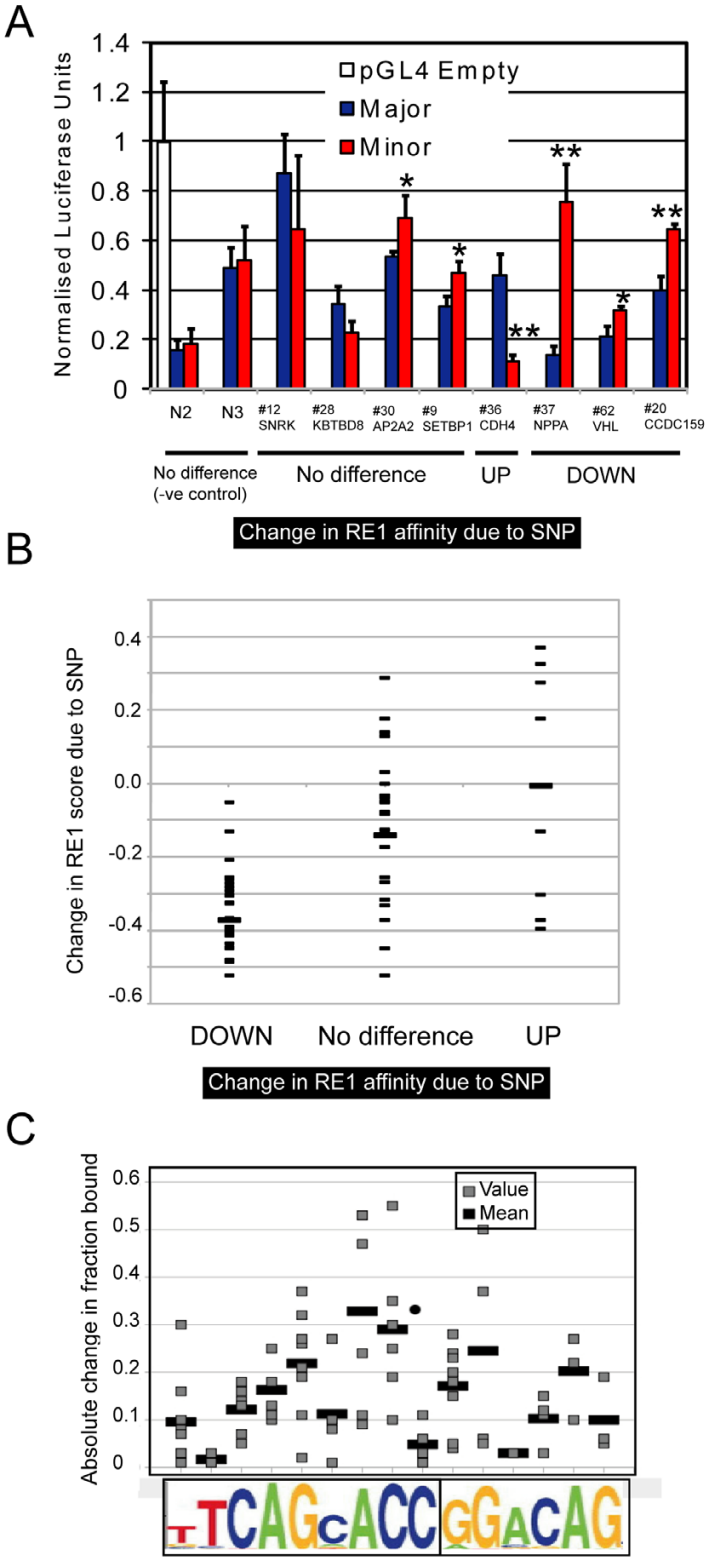
Validation and analysis of RE1 SNPs. (A) Luciferase reporter gene assay was used to test the function of pRE1 sequences. Data were normalised to results from an “Empty” pGL4TK reporter gene plasmid containing no RE1 sequence. On the x-axis, RE1s are grouped according to their performance in the EMSA assay. We included two control RE1 pairs (N2, N3) having SNPs outside their core RE1 sequence. In each case the activity of Major and Minor RE1 alleles were tested in parallel and compared by Student's *t* test (* P
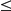
0.05, ** P
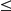
0.01). Firefly luciferase values were normalised to cotransfected Renilla luciferase, and values are expressed relative to the empty pGL4TJ plasmid. (B) We estimated the change in the quality of each RE1 motif resulting from the presence of a SNP, using a position specific scoring matrix [19] (shown on the y-axis). All RE1s in this study are grouped according to their performance in EMSA. The mean of each set is shown by a wider bar. (C) The absolute change in affinity resulting from a SNP is plotted, as a function of the SNP location within the RE1 motif (grey bars). The absolute fold change was found by calculating the difference between the Fraction Bound values for Major and Minor RE1 alleles. For each location, the mean value is shown by a wider bar.

Several lines of evidence suggest that our EMSA method is accurate in predicting the outcome of a SNP variant within an RE1 element. First, we validated selected SNPs using luciferase assay, which measures the ability of an RE1 to repress gene transcription of a reporter gene (Figure 4A). Consistent with REST functioning as a transcriptional repressor, SNPs that increased the affinity for REST (“UP”), increased the repression of the luciferase reporter gene, and vice versa (“DOWN”). We did not observe robust differences in reporter activity when comparing RE1 alleles that do not differ in affinity, as gauged by EMSA (“No difference”).

Motif analysis of the pRE1s also supports the EMSA assay results. Transcription factor binding to DNA is guided by the presence of a particular sequence element, and the affinity of binding is generally in proportion to the similarity that the element has to an idealised motif [24]. Thus, single nucleotide changes that make an element more similar to the ideal motif are expected to increase the element's affinity, and vice versa. We tested this, by using the RE1 PWM to estimate changes in pRE1 motif quality due to the presence of a SNP (Figure 4B). We divided the RE1s between those where the minor allele had greater affinity (“UP”), weaker affinity (“DOWN”), or no difference. These data show that the effect of a SNP on binding affinity agrees well the expectation based on motif analysis: SNPs that strongly decrease the RE1 motif quality, also strongly decrease the RE1 binding affinity, and vice versa. We also observed diversity in the strength of the effect of SNPs depending on their location within the RE1 motif (Figure 4C).

Finally, the degree of in vivo binding to these RE1 sites, as measured by ChIPseq, correlates with the degree of binding observed in the EMSA assay (Figure S1). We used REST ChIPseq data from the ENCODE consortium to measure the degree of in vivo binding of REST to pRE1s, and compared this to the binding affinity discovered by EMSA. This analysis showed that REST recruitment in vivo is significantly correlated with the RE1 motif's affinity, at least in GM12878 cells.

**Figure 5:**
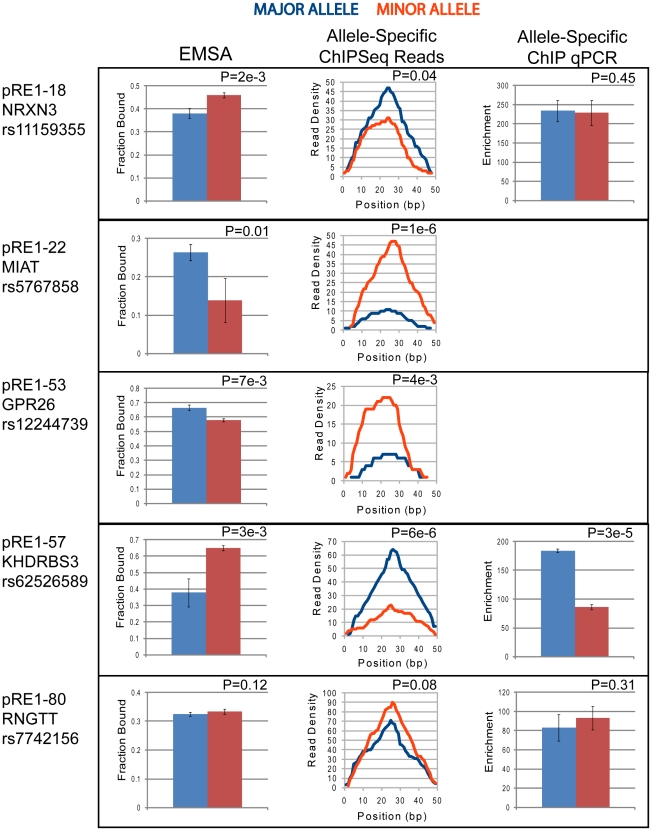
Allele-specific recruitment to pRE1s in GM12878 cells. The data shown corresponds to the 5 heterozygous SNPs discovered in GM12878 cells. In all cases blue indicates the Major allele, and red the Minor allele. EMSA data is shown in left panel (Note that Fraction Bound units correlate inversely with binding affinity), allele-specific ChIPseq read density from the ENCODE project is shown in the central panel, and allele-specific ChIP enrichment (where available) is shown in the right panel. Statistical significance was calculated using Student's *t* test (EMSA, allele-specific ChIP) and Binomial statistics (ChIPseq).

Clearly, it is important to demonstrate that changes in RE1 affinity due to observed SNPs actually alter the recruitment of REST in the cell nucleus. It has been shown that differential affinity of TFBS variants can be identified using allele-specific quantitative PCR and Sanger sequencing methods [9]. We reasoned that, in a similar way, allele-specific reads in high throughput ChIPseq should reflect a bias in allele affinity. Using data from the ENCODE project for GM12878 cells, we searched for sequencing reads specifically mapping to one or other pRE1 alleles, and tested for statistically significant differences in the frequencies of reads. We chose these cells since, unlike the majority of cell lines, they are karyotypically normal and genetic abornamalities will not confound the analysis of allele-specific sequencing reads. As expected, the GM12878 cells are homozygous for most of the pRE1s - as evidenced by a complete absence of read matches representing the 25mers overlapping the minor SNP sequence variant. In the five cases shown in Figure 5, non-zero numbers of minor allele SNP matches were observed, indicating that the pRE1s are heterozygous in these cells. The allelic read frequencies in all cases agree with the changes in affinity reported by EMSA, indicating that the in vitro affinity measurements are a good representation of the effect that a SNP has on genomic recruitment of REST. In one case, pRE1-80, the EMSA showed that the SNP has no effect on affinity, and this is reflected in no significant difference in ChIPseq reads between the two alleles. Three lines of evidence support the accuracy of this analysis: First, we repeated the analysis for the second independent NRSF Chipseq performed by ENCODE in GM12878 cells, and observed the same allelic biases (Figure S2). Second, we repeated this analysis on another karyotypically normal cell line in the ENCODE dataset - the human H1 embryonic cell line - and again observed allelic ChIPseq biases that were consistent with predictions by the EMSA assay in two out of three cases (in one case, the allelic ChIPseq reveals increased affinity of the minor allele of pRE1-53, which is also observed in EMSA but below the threshold we imposed) (Figure S3). Finally, we experimentally validated these findings in the GM12878 cells using allele-specific ChIP, interrogated by quantitative PCR (Figure 5). We managed to design primers specific to major and minor pRE1 alleles in three cases, and could confirm strongly increased binding of REST to the major allele of pRE1-57. We could detect no difference in binding between the alleles of pRE1-18 and pRE1-80, which broadly reflects the results of EMSA/ChIPseq, where no substantial difference in affinity was found.

In the course of this study, two important datasets were released: REST (NRSF) ChIPseq data for multiple cell lines from the ENCODE consortium [25], and updated dbSNP polymorphism catalogues incorporating data from the 1000Genomes project [26]. We have used this data to compile a second-generation set of polymorphic RE1s, which can be found in Methods S1 and File S5. Based on lessons learned in the first attempt, we modified our analysis pipeline in several ways: RE1 motifs were scanned at low score threshold, to improve discovery of pRE1s with higher-affinity minor alleles; motifs were filtered through ENCODE ChIPseq binding regions combined from five cell lines to improve the sensitivity of motif discovery; and we only considered common SNPs (at least 1% frequency), to avoid the discovery of extremely rare or artifactual SNPs such as rs12565 in *NPPA*. Details of this analysis can be found in Methods S1. Altogether, this second-generation analysis yielded 628 SNPs overlapping 601 candidate RE1s. These will form the basis of future, more sensitive analysis of polymorphic RE1s.

## Discussion

The functional annotation of phenotypic human polymorphisms is presently a serious bottleneck that prevents advances in genomics from being translated into new therapies. Thus it is imperative that, in parallel to gathering genome-wide association data, efforts be made to annotate regulatory SNPs genome-wide. This has been recognised and attempted by a number of groups [27], [28], [29], [9]. By demonstrating that regulatory polymorphisms can be identified and characterised, we have shown that such annotation is feasible, and allows a clear mechanistic explanation linking noncoding DNA polymorphism with known genomic regulatory pathways and likely disease roles.

We have shown that the genomic motifs targeted by REST differ beween individual humans as a result of genetic polymorphisms. These polymorphic binding sites have different biochemical affinities for REST, resulting in altered in vivo transcription factor recruitment and differential reporter gene repression. This results in clear and consistent changes in in vivo binding of REST, as evidenced by our analysis of allele-specific ChIPseq reads.

The levels or activity of REST has been shown to contribute to various pathologies [30]. For example, in Huntington's disease, excessive levels of REST are found in the nucleus, repressing target genes such as *BDNF* and resulting in neurodegeneration [31]. In contrast, loss of functional REST expression through genetic mutations has been shown to contribute to colon cancer progression [13]. Coupled with the fact that we have identified SNPs that can either increase or decrease pRE1 affinity, these findings suggest that pRE1s may either increase or decrease the likelihood and/or severity of associated diseases. Clearly the next step is to interrogate the frequency of pRE1 SNPs in genome-wide association studies (GWAS) for REST-associated diseases, to validate that they do indeed contribute to disease phenotypes as predicted. This analysis has been hindered by the present lack of availability of raw GWAS data due to patient confidentiality issues, meaning that raw data from published studies is not required to be freely shared, in contrast to the norm for other types of genomic data.

The pRE1s identified in this study are often located in the vicinity of genes involved in diseases linked to REST mediated mis-regulation, most notably cardiovascular disease, neurological diseases and cancer (Tables 1 and 2, and File S1). Amongst the most promising pRE1 candidates we identified was that in the pRE1-37, residing in the 3′UTR of the *NPPA* gene. This encodes the atrial natriuretic peptide, a crucial signalling peptide whose circulating levels are strongly correlated with blood pressure [32]. Regulation of this gene by REST is a major pathway in cardiac hypertrophy: loss of REST expression in diseased heart allows the re-activation of the fetal cardiac gene expression program and resultant hypertrophy [15]. A recent large scale study identified a nearby SNP (rs5068) in the *NPPA* 3′UTR as the single strongest predictor within that locus of circulating ANP levels [32]. We initially thought that the SNP in pRE1-37 (rs12565) - that was not directly interrogated in the Newton-Cheh study - was the true causative variant and was in linkage disequilibrium with rs5068. However, subsequent genotyping in the Hapmap CEU cohort indicated that these two SNPs are not linked (File S3). In fact, we did not detect any instances of rs12565 in the 30 CEU individuals, suggesting that it is rare. Given that the SNP reduces the affinity of the RE1, and that REST is known to repress ANP levels within the cardiovasculature [15], we may surmise that the minor variant confers a tedency towards reduced blood pressure on carriers. It will be exciting to observe the results of future deeper genotyping assays to reveal the true frequency of this polymorphism.

Our experimental method had a number of deficiencies that may be improved in future. First, our initial scan for RE1 motifs was performed on the reference genome, meaning that it was biased towards discovering Major allele RE1s. Second, allelic affinity differences were only assayed in EMSA at a single competitor concentration which may lead to false negatives when the free energy of binding of the allelic pair is very different from the idealized probe. Having detailed biophysical binding models and measuring actual binding energies would increase the accuracy of the screen, exemplified by the automated fluorescence anisotropy method employed by Fersht and colleagues [33]. Third, we only assayed for the presence of polymorphisms in the minority of REST-bound genomic loci where recruitment can unambiguously be ascribed to a high-quality, full-length RE1 element. Hopefully, future studies will shed light on the cryptic processes mediating recruitment to the other 52% of loci containing single half sites, or no identifiable motif at all [21], [10]. Finally, many other types of genetic polymorphism exist in addition to SNPs, the effects of which on REST targeting we have not examined, but is likely to be important.

One important weakness in this study that only became apparent subsequently, can be inferred from Figure 4B. While there is a clear trend for SNPs that decrease the RE1 motif score to also strongly decrease their binding affinity, this relationship is not strong for SNPs that *increase* binding affinity. This is likely to be result of the way we designed our analysis: We only searched for high quality motifs in the reference genome. Therefore we specifically selected against RE1s that have a weak motif in the major allele, that increases in the minor allele. Those RE1s in the present study that have higher affinity in the minor allele, are likely to be outliers that are in fact poorly modelled by the RE1 PWM. The other RE1s that would behave in the expected way (increase binding affinity, increase in PWM score), are specifically selected against, because they have a motif score below cutoff in the reference genome, and thus were exluded from our analysis. This weakness of our design strategy will be addressed in future.

Recent technological progress in microarray and sequencing technologies has enabled rapid advances in human genetics, specifically in (a) the gathering of genome-wide association data, and (b) the depth of annotation of human genetic variants. The latter refers specifically to the ongoing 1000Genomes Project, which is set to revolutionise human SNP catalogues. During preparation of this manuscript, the numbers of SNPs in dbSNP has increased by 61% (23653737 in dbSNP131 vs 14708752 in dbSNP129), and we thus expect that future studies will identify similarly more pRE1s. In particular, SNPs from the 1000Genomes project are likely to include many rarer variants that are not represented in current annotations, and are likely to include strongly-acting disease variants that remain undetected at present and are posited to underlie much of the currently unexplained genetic basis of common diseases. This means that both the number of phenotype-associated SNPs *and* the total number of known SNPs are likely to increase rapidly in the future. As a consequence, regulatory SNP annotations will quickly become obsolete, and will need to be repeated as genetic catalogues grow. To account for this, we used the latest data available from ENCODE and dbSNP to create a second generation pRE1 dataset, now comprising 628 SNPs overlapping 601 candidate pRE1s. It is likely that these numbers will continue to increase in the near future. We anticipate that the combination of genetics and genomics such as that presented in this study will yield powerful insights into the numerous polymorphisms that, in combination, are likely determine the unique disease susceptibility of each individual.

## Materials and Methods

### Discovery of SNPs in RE1 motifs

All genomic coordinates in this study are based on human genome version hg18. ChIPseq data for REST is based on the 1946 binding peaks discovered previously in human Jurkat cells [10]. The locations of binding peaks were converted from human genome version hg17 to hg18 using the UCSC LiftOver tool. Because the binding regions were of heterogeneous size, we extended/reduced the size of each region to 100 bp centred on the binding peak. We used the position weight matrix (PWM) search program Seqscan [19] to identify RE1 motifs in the 100 bp ChIPseq regions. In order to include RE1 motifs with non-canonical spacer lengths, motif searches were carried out using PWMs representing all possible spacer lengths from 0 to 12 bp (the canonical RE1 contains a spacer of 2 bp). In each case, the single highest scoring RE1 was assumed to account for REST binding. As a result, we found 942 ChIPseq regions that contained an RE1 with a score of 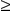
0.88 from Seqscan, corresponding to a medium stringency motif quality that is not found in appreciable numbers in random genomic DNA. We reasoned that SNPs in the most strongly constrained region of the RE1 motif would be most likely to disrupt REST binding. Thus we separated all RE1s into their left and right half sites (corresponding to positions 1–9 and 12–17 in the canonical motif), and overlapped these locations with all SNPs of length 1 from dbSNP129, using the Galaxy package [34]. We considered the sequence variant that occurs in the human reference genome (and usually the more frequent variant) to be the Major allele, and the less frequent sequence variant to be the Minor allele.

### Electrophoretic mobility shift assay for comparison of RE1 alleles

EMSAs were carried out as described previously using a 30 bp double stranded RE1 element probe modified with 5′ Cy5 label on both strands, adapted from the rat *Scn2a2* gene [35], [20]. Probe was premixed with various unlabelled competitor DNA sequences at a molar ratio of competitor∶probe 300∶1, at 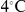 for 1 h. A master mix containing purified recombinant HisMBP-REST/DBD protein was then added to the premixed DNA, such that the final reaction mixture contained 0.5 nM probe, 150 nM competitor DNA and 40 nM protein in EMSA buffer (2 mM 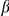 -mercaptoethanol, 10 mM TrisHCl pH 8.0, 100 mM KCl, 50 M ZnCl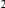, 10% ultra pure glycerol, 0.1% NP-40, and 0.1 mg/ml bovine serum albumin). This mixture was incubated at 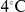 for 1 h. Unlabelled rat *Chrm4* RE1 was used as a positive control competitor (validated high affinity RE1)(“Ideal”). A mutated version of this sequence was used as non-specific dsDNA control competitor (“Mut”). The bound and unbound probes were subsequently separated at 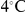 on a pre-run tris-glycine 12% polyacrylamide gel at 200 V for 30 min. The fluorescence was detected using a Typhoon 9140 PhosphorImager (Amersham), and bound and unbound probe bands were quantified using ImageQuant 5.2 software (Amersham). For every competitor DNA, three independent reaction mixes were made and run on the same gel, and all error bars and statistical tests were carried out using these three replicates. Statistical tests were carried out using Student's *t* test (unpaired, one-sided, not corrected for multiple testing). All quantitation data is available in File S2 and all raw EMSA images are available from the Authors upon request.

### Luciferase transcription assay

Forty base pair oligonucleotides encompassing candidate RE1s were cloned into the pGL4 luciferase reporter plasmid (Promega), upstream of a thymidine kinase (TK) promoter, using BglII and KpnI restriction sites. HEK293 cells were maintained at 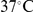 in 5% CO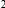 with Dulbecco's modified Eagle's medium (DMEM), supplemented with 10% fetal bovine serum, 4500 mg/L glucose, 4 mM L-glutamine, 100 units/ml penicillin and 100 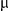
g/ml streptomycin. Cells were seeded at a density of 7.5×10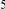 cells per well in 24-well plates the day before transfection. For each well, 100 ng luciferase reporter plasmids and 5 ng pRL Renilla transfection control plasmid were mixed and transfected using Lipofectamine 2000 (Invitrogen) following the manufacturers protocol. After 48 hours, cells were harvested and the reporter activity was analyzed using Dual Luciferase Reporter Assay (Promega) and GloMax-Multi+ chemiluminescence microplate reader (Promega) according to the manufacturers instructions. Three biological replicates were carried out in two technical replicates each. Luciferase activities were normalized to the corresponding Renilla readings, then divided by the background activity as measured for pGL4-TK plasmids lacking the RE1 insert.

### Allelic analysis of ChIPseq reads

Raw ChIPseq reads were downloaded from the ENCODE dataset within UCSC genome browser in FASTQ format. We employed the recently released ENCODE dataset for REST (also known as NRSF) in GM12878 (Epstein-Barr virus transformed lymphoblastoid cells from one of the Hapmap cohort) (25 bp reads) and H1 (human embryonic stem cells) (35 bp reads) because (a) these datasets has a higher number of sequencing reads than previous ChIPseq studies on REST [10], and (b) because these cells have normal karyotype. One can only distinguish between reads from polymorphic regions containing a SNP, by inspection of the sequencing reads that directly overlap that SNP. Therefore, we used a custom script to generate all possible reads that uniquely and exactly overlap each pRE1 SNP, that distinguish Major and Minor variants. We considered a pRE1 to be heterozygous in a given cell line, if it had at least 5 reads overlapping both major and minor variants. In these cases, we compared the numbers of each variant read using the exact binomial test in R to generate statistical significance. We checked for heterozygous pRE1 SNPs that could have another nearby SNP (within one ChIPseq read length) that could confound this analysis: we identified a single case, rs28396985, which was omitted from further analysis.

### Allelic-specific ChIP

GM12878 cells were grown in RPMI media supplemented with 15% FBS and 1 mM L-glutamine. Cells were cross-linked by adding formaldehyde directly to the media at a final concentration of 1%. Cross-linking was quenched after 10 minutes by adding glycine at a final concentration of 200 mM. Nuclei were obtained by washing cells in cell lysis buffer (10 mM Tris pH 8, 0.25% Triton-X 100, 10 mM EDTA, 0.1 M NaCl). Nuclei were lysed in high SDS lysis buffer (50 mM Hepes pH 7.5, 150 mM NaCl1, 2 mM EDTA, 1% Triton X-100, 0.1% Na deoxycholate, 1% SDS) and chromatin was washed twice in low SDS lysis buffer (50 mM Hepes pH 7.5, 150 mM NaCl1, 2 mM EDTA, 1% Triton X-100, 0.1% Na deoxycholate, 0.1% SDS). Chromatin was sonicated to an average size of 300 bp and immuno-precipitation was performed using REST-specific antibody (Santa Cruz). Immunoprecipitated DNA was eluted, reverse-crosslinked and purified by phenol∶chloroform extraction. Enrichment of RE1s was read out using quantitative real-time PCR with allele-specific primers. Control ChIP experiments are shown in Figure S4, and allele-specific primer sequences in File S4. We designed two primer pairs per SNP, one for each allele (Major and Minor). One oligo in each pair was designed such that its 3′end coincided with the SNP. To increase specificity, a mismatch was introduced in the penultimate position. The other primer did not coincide with the SNP nucleotide and was shared between the pairs. To calculate enrichment for the ChIP-enriched DNA, we normalized the Ct values obtained for the SNP-specific product with the Ct values obtained from amplifying a locus in a GAPDH exon (non-target control) to generate Delta Ct values. These were further normalized to Input (non-immunoprecipitated DNA), and fold enrichment was calculated by the DeltaDelta Ct method.

## Supporting Information

Figure S1 Correlation of in vivo recruitment to in vitro affinity of pRE1s.Using raw ENCODE ChIPseq reads, binding peaks were recovered using MACS [36] at default settings. Relevant control Input libraries were used for normalisation. ChIPseq enrichment values for each pRE1 were plotted against the relative binding from EMSA - defined as (1 - Fraction Bound). The non-parametric Spearman method was used to compute the correlation between Enrichment and Fraction Bound. This correlation is expected to be negative, since the value of Fraction Bound decreases with the increasing binding. Statistical significance of these correlations is also shown. Two available replicates of each of GM12878 and H1 cell lines were analysed independently.
(PDF)

Figure S2 Allele-specific ChIPseq reads in two independent biological replicates. Identical analysis was carried out on the two GM12878 NRSF (REST) ChIPseq libraries available from the ENCODE consortium. Charts show the number of uniquely mapping reads originating from Major or Minor alleles of pRE1s found to be heterozygous in GM12878. The data in the left panel (Replicate 1) correspond to those shown in Figure 5 of the main text.
(PDF)

Figure S3 Allelle-specific ChIPSeq analysis in H1 cell line. Using data from the ENCODE consortium, we extracted those sequencing reads mapping uniquely and specifically to all pRE1s. We identified three heterozygous cases having non-zero reads for both major and minor alleles, shown here. The figures show the density of reads at each position around the relevant SNP for Major (blue) and Minor (red) alleles. Left panel shows EMSA data (Note that units are in Fraction Bound, which is inversely correlated to binding affinity), right panel shows ChIPseq read density. Statistical significance was calculated using Student?s t test (EMSA) and Binomial statistics (ChIPseq).
(PDF)

Figure S4 Control experiments for allel-specific ChIP. Shown are enrichment values for conventional ChIP carried out using an anti-REST antibody in GM12878 cells. *ACTB* and *RPL* amplicons are not proximal to any REST binding site, and thus are not expected to show enrichment. Data is also shown for conventional primer sets (ie not allele-specific) to pRE1s indicated, where REST is expected to be recruited.
(PDF)

File S1 Complete list of polymorphic RE1s identified in this study (First Generation pRE1 Catalogue).
(XLS)

File S2 Raw EMSA quantification data.
(XLS)

File S3 Genotyping of pRE1s in CEU Hapmap population.
(XLS)

File S4 ChIP qPCR primer sequences.
(DOC)

File S5 Complete list of polymorphic RE1s identified in the Second Generation pRE1 Catalogue.
(XLS)

Methods S1 Description of the Second-Generation annotation of polymorphic RE1s.
(DOC)
