# Supplementary material for: A Genome-Wide Screen for Genetic Variants That Modify the Recruitment of REST to Its Target Genes
Source: PLoS Genet. 2012 Apr 5;8(4):e1002624. doi: 10.1371/journal.pgen.1002624 (PMC3320604; doi:10.1371/journal.pgen.1002624)

Supplementary Figure S1

Johnson et al.,
A Genome-wide Screen for Genetic Variants that Modify the Recruitment of REST to its Target Genes

Replicate 1
Spearman Rank Correlation -0.397
P=8.74EXP-4

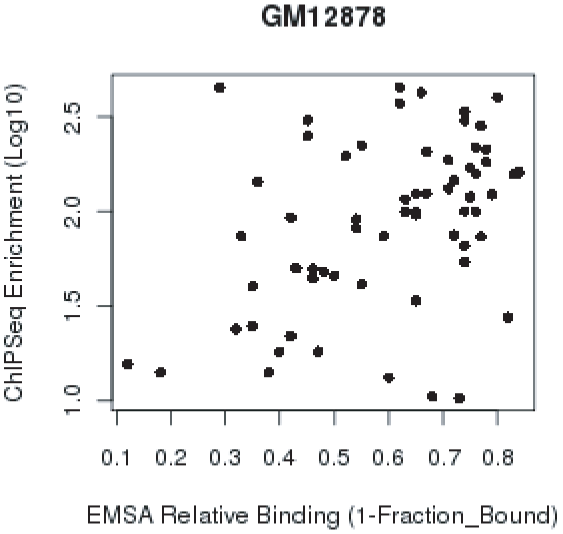

Replicate 2
Spearman Rank Correlation -0.391
P=0.0015

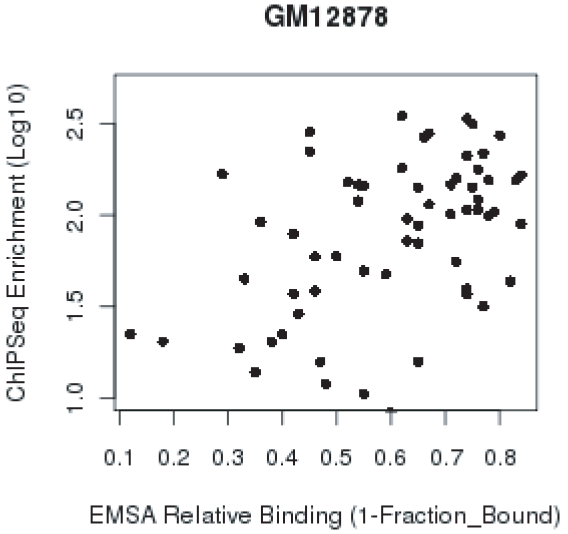

Replicate 1
Spearman Rank Correlation -0.235
P=0.047

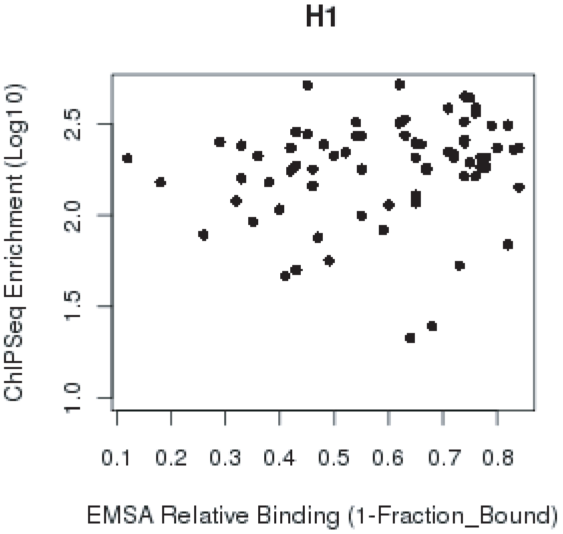

Replicate 2
Spearman Rank Correlation -0.142
P=0.233

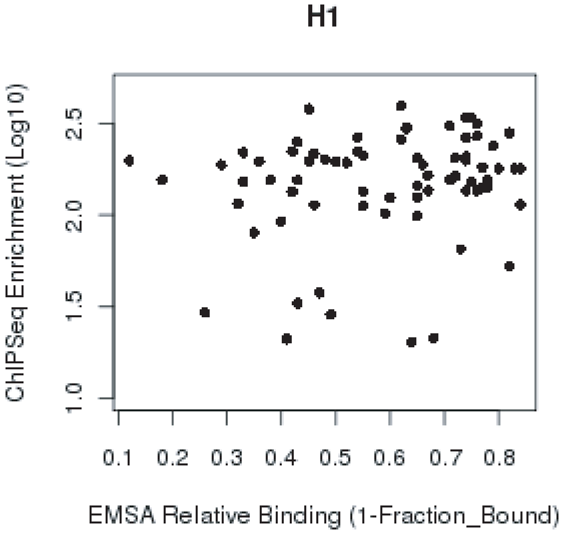

Supplement: Figure S1 — Correlation of in vivo recruitment to in vitro affinity of pRE1s.Using raw ENCODE ChIPseq reads, binding peaks were recovered using MACS [36] at default settings. Relevant control Input libraries were used for normalisation. ChIPseq enrichment values for each pRE1 were plotted against the relative binding from EMSA - defined as (1 - Fraction Bound). The non-parametric Spearman method was used to compute the correlation between Enrichment and Fraction Bound. This correlation is expected to be negative, since the value of Fraction Bound decreases with the increasing binding. Statistical significance of these correlations is also shown. Two available replicates of each of GM12878 and H1 cell lines were analysed independently. (PDF) [file pgen.1002624.s001.pdf]
