# Supplementary material for: A Genome-Wide Screen for Genetic Variants That Modify the Recruitment of REST to Its Target Genes
Source: PLoS Genet. 2012 Apr 5;8(4):e1002624. doi: 10.1371/journal.pgen.1002624 (PMC3320604; doi:10.1371/journal.pgen.1002624)

Supplementary Figure S2

Johnson et al.,
A Genome-wide Screen for Genetic Variants that Modify the Recruitment of REST to its Target Genes

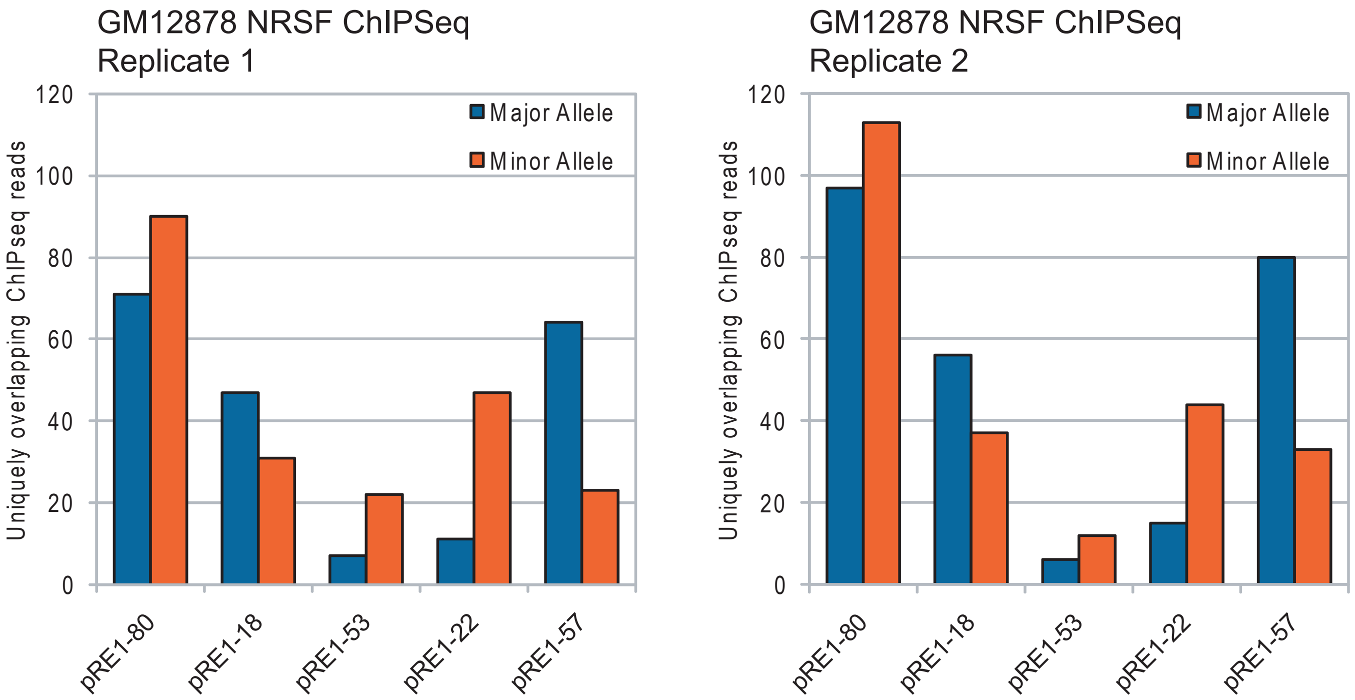

Supplement: Figure S2 — Allele-specific ChIPseq reads in two independent biological replicates. Identical analysis was carried out on the two GM12878 NRSF (REST) ChIPseq libraries available from the ENCODE consortium. Charts show the number of uniquely mapping reads originating from Major or Minor alleles of pRE1s found to be heterozygous in GM12878. The data in the left panel (Replicate 1) correspond to those shown in Figure 5 of the main text. (PDF) [file pgen.1002624.s002.pdf]
