# Supplementary material for: A Genome-Wide Screen for Genetic Variants That Modify the Recruitment of REST to Its Target Genes
Source: PLoS Genet. 2012 Apr 5;8(4):e1002624. doi: 10.1371/journal.pgen.1002624 (PMC3320604; doi:10.1371/journal.pgen.1002624)

Supplementary Figure S3

Johnson et al.,
A Genome-wide Screen for Genetic Variants that Modify the Recruitment of REST to its Target Genes

H1 ES Cells

rs11159355
pRE1-18
NRXN3

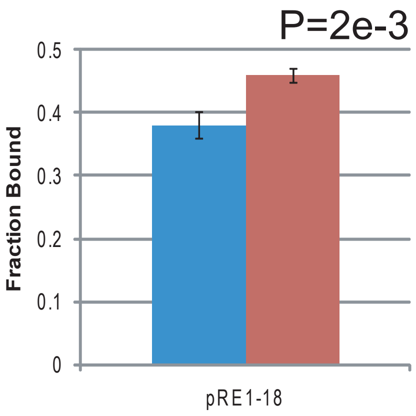

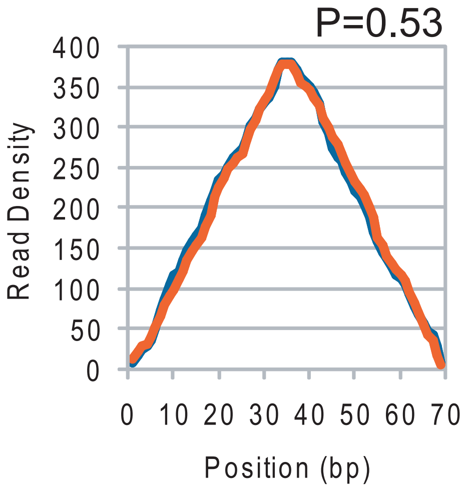

rs12244739
pRE1-53
GPR26

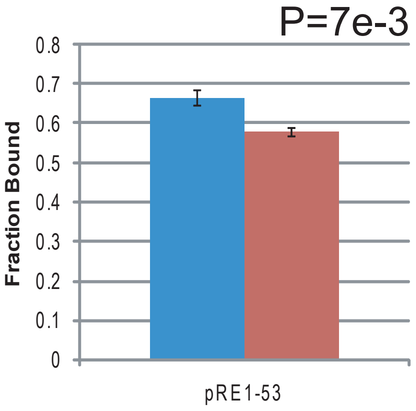

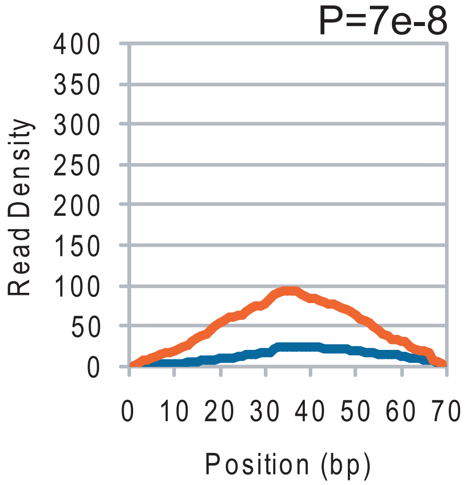

rs3979
pRE1-56
ADRA2C

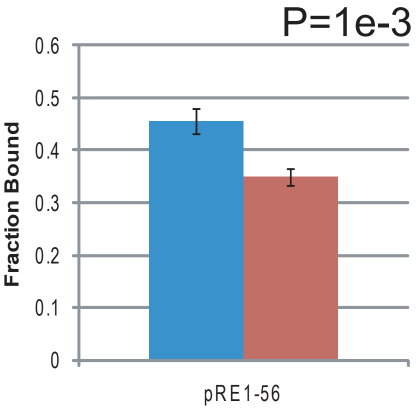

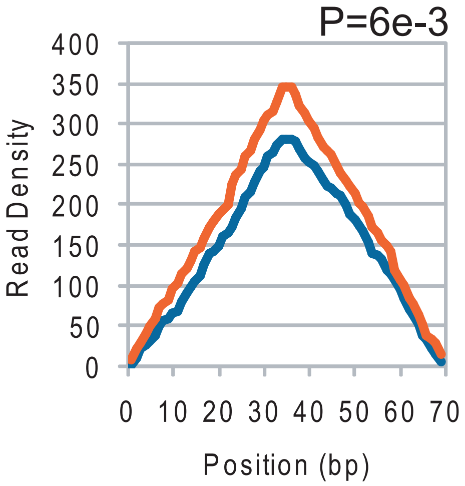

Supplement: Figure S3 — Allelle-specific ChIPSeq analysis in H1 cell line. Using data from the ENCODE consortium, we extracted those sequencing reads mapping uniquely and specifically to all pRE1s. We identified three heterozygous cases having non-zero reads for both major and minor alleles, shown here. The figures show the density of reads at each position around the relevant SNP for Major (blue) and Minor (red) alleles. Left panel shows EMSA data (Note that units are in Fraction Bound, which is inversely correlated to binding affinity), right panel shows ChIPseq read density. Statistical significance was calculated using Student?s t test (EMSA) and Binomial statistics (ChIPseq). (PDF) [file pgen.1002624.s003.pdf]
