# Supplementary material for: A Genome-Wide Screen for Genetic Variants That Modify the Recruitment of REST to Its Target Genes
Source: PLoS Genet. 2012 Apr 5;8(4):e1002624. doi: 10.1371/journal.pgen.1002624 (PMC3320604; doi:10.1371/journal.pgen.1002624)

Supplementary Figure S4

Johnson et al.,
A Genome-wide Screen for Genetic Variants that Modify the Recruitment of REST to its Target Genes

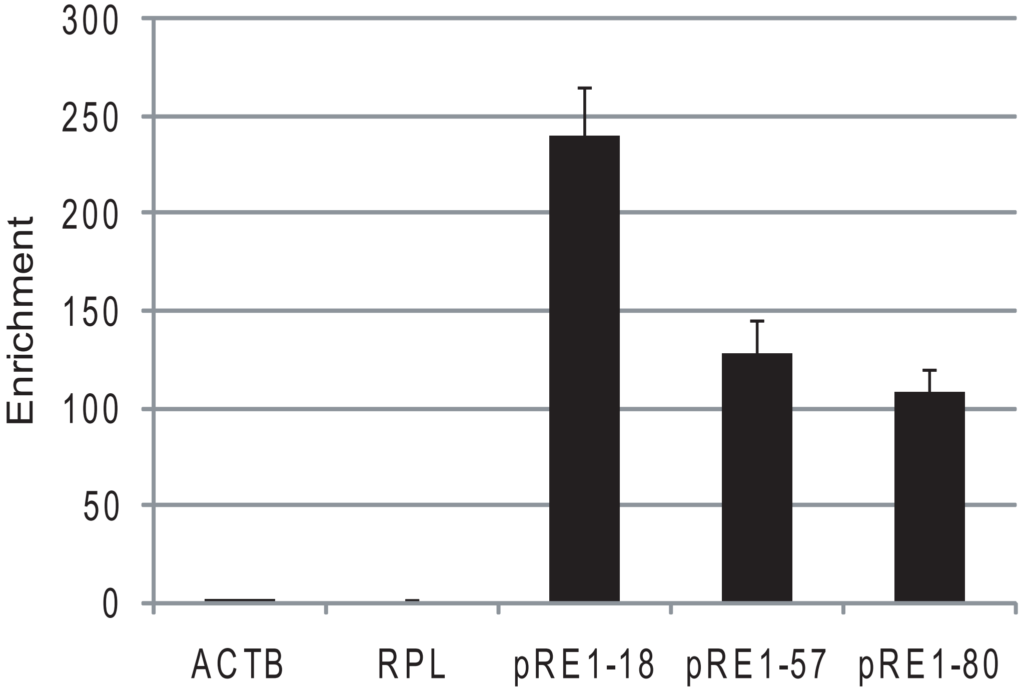

Supplement: Figure S4 — Control experiments for allel-specific ChIP. Shown are enrichment values for conventional ChIP carried out using an anti-REST antibody in GM12878 cells. ACTB and RPL amplicons are not proximal to any REST binding site, and thus are not expected to show enrichment. Data is also shown for conventional primer sets (ie not allele-specific) to pRE1s indicated, where REST is expected to be recruited. (PDF) [file pgen.1002624.s004.pdf]
