# Supplementary material for: A Genome-Wide Screen for Genetic Variants That Modify the Recruitment of REST to Its Target Genes
Source: PLoS Genet. 2012 Apr 5;8(4):e1002624. doi: 10.1371/journal.pgen.1002624 (PMC3320604; doi:10.1371/journal.pgen.1002624)
Supplement: File S4 — ChIP qPCR primer sequences. (DOC) [file pgen.1002624.s008.doc]

Supplementary File S4

Johnson et al.,

A Genome-wide Screen for Genetic Variants that Modify the Recruitment of REST to its Target Genes

Primers for ChIP-PCR.

| SNP ID | Primer sequence | Detected allele |
| --- | --- | --- |
| rs7742156 | AGGTGTCATGCTTCAGTTTC  TCTCCCACATTCAGAGGCTCT | Major (A) |
|  | AGGTGTCATGCTTCAGTTTC  TCTCCCACATTCAGAGGCTCC | Minor (G) |
|  | | |
| rs11159355 | GGCTATTTTGGAAATACGAAGG  TGAGAGCTTTAAGGAGAGTCCAAC | Major (G) |
|  | GGCTATTTTGGAAATACGAAGG  TGAGAGCTTTAAGGAGAGTCCAAT | Minor (A) |
|  | | |
| rs62526589 | TGAATTCCCTTTCAGCAGTATG  ACAAGGGAGCTGTCCAGCAG | Major (C) |
|  | TGAATTCCCTTTCAGCAGTATG  ACAAGGGAGCTGTCCAGCAT | Minor (A) |
|  | | |
| rs5767858 | GCCATCTGTGAGCCAGAAAG  ATTCTGGAGGCTGGAAAGGC | Major (G) |
|  | GCCATCTGTGAGCCAGAAAG  ATTCTGGAGGCTGGAAAGGT | Minor (A) |
|  | | |
| rs12244739 | CCAGCACTGGTCTGCATAC  GCAGAGATCAAGGCAAGGAG | Major (C) |
|  | CCAGCACTGGTCTGCATAT  GCAGAGATCAAGGCAAGGAG | Minor (T) |
|  | | |
| Rpl19 | TCTCCACAGGTAAGCGGAAG  TGACAAAGTGGTCGTTGAGG |  |
|  | | |
| Gapdh | ACCAGGTGGTCTCCTCTGAC  TGACAAAGTGGTCGTTGAGG |  |
