# Supplementary material for: A Genome-Wide Screen for Genetic Variants That Modify the Recruitment of REST to Its Target Genes
Source: PLoS Genet. 2012 Apr 5;8(4):e1002624. doi: 10.1371/journal.pgen.1002624 (PMC3320604; doi:10.1371/journal.pgen.1002624)
Supplement: Methods S1 — Description of the Second-Generation annotation of polymorphic RE1s. (DOC) [file pgen.1002624.s010.doc]

Supplementary Methods S1

Johnson et al.,

*A Genome-wide Screen for Genetic Variants that Modify the Recruitment of REST to its Target Genes*

Description of the second-generation dataset of polymorphic RE1s.

Since the original analysis performed in this project, deeper genetic and genomic data has become available. Therefore, we have repeated the curation of pRE1s using more recently available ChIPseq (from the ENCODE consortium) and SNP (from dbSNP135) data. The final dataset is made available in Supplementary File 10.

We scanned the human genome build GRCh37 / hg19 using the Seqscan program with RE1 PWMs, having a range of spacer sizes from 0-12 (REF). We used a relaxed score threshold of 0.87. This yielded altogether 317,138 candidate motifs.

We next used recent ENCODE ChIPseq data to filter the RE1 motifs. We downloaded ChIPseq peak location BED format files (Replicate 1 in all cases) from the UCSC Genome Browser for the following cell types: GM12878, H1, HeLaS3, HepG2, K562. Given that many peaks will be shared between different cell types, we merged the binding locations BED coordinates, using the Galaxy web tool (Giardine et al, PMID 16169926). This yielded 54,934 non-overlapping binding regions.

The candidate RE1 motifs were filtered such that we only retained those that overlapped a ChIPseq binding region by at least 1 bp, resulting in 15,155 experimentally-supported motifs. All such intersections were carried out using the IntersectBED functionality within the BEDTools suite (Quinlan et al, PMID 20110278). The RE1 motif consists of two strongly conserved “half sites” (position 1-9, and 12-17) that are important for binding affinity, in addition to the spacer and 3' regions, which are not important for binding. We only considered SNPs overlapping or falling within the half sites.

Finally, human SNPs were overlapped with the set of RE1 half sites. The complete set of “common” (at greater than 1% frequency in the population) human SNPs (dbSNP135) were downloaded from UCSC Genome Table Browser (11,525,489). Using IntersectBED, we found altogether 628 SNPs overlapping 601 distinct RE1s (601 / 15,155 = 4.0%).

We used the GREAT tool (McLean et al, PMID 20436461) to predict the target genes of these RE1 sites with the following settings: GREAT version 1.8, Association rule: Basal+extension: 5000 bp upstream, 1000 bp downstream, 1000000 bp max extension, curated regulatory domains included.
